# LKB1 signaling is altered in skeletal muscle of a Duchenne muscular dystrophy mouse model

**DOI:** 10.1242/dmm.049930

**Published:** 2023-07-10

**Authors:** Brigida Boccanegra, Paola Mantuano, Elena Conte, Alessandro Giovanni Cerchiara, Lisamaura Tulimiero, Raffaella Quarta, Erika Caputo, Francesca Sanarica, Monica Forino, Valeria Spadotto, Ornella Cappellari, Gianluca Fossati, Christian Steinkühler, Annamaria De Luca

**Affiliations:** ^1^ Department of Pharmacy-Drug Sciences, University of Bari ‘Aldo Moro’, 70125 Bari, Italy; ^2^ Preclinical R&D Department, Italfarmaco S.p.A., Cinisello Balsamo, 20092 Milan, Italy

**Keywords:** Duchenne muscular dystrophy, LKB1, AMPK, Epigenetic, Muscle metabolism, Mouse models

## Abstract

The potential role of liver kinase B1 (LKB1) in the altered activation of the master metabolic and epigenetic regulator adenosine monophosphate-activated protein kinase (AMPK) in Duchenne muscular dystrophy has not been investigated so far. Hence, we analyzed both gene and protein levels of LKB1 and its related targets in gastrocnemius muscles of adult C57BL/10 *mdx* mice and D2 *mdx* mice, a model with a more severe dystrophic phenotype, as well as the sensitivity of the LKB1–AMPK pathway to AMPK activators, such as chronic exercise. Our data show, for the first time, a reduction in the levels of LKB1 and accessory proteins, MO25 and STRADα, in both *mdx* strains versus the respective wild type, which was further impaired by exercise, in parallel with a lack of further phosphorylation of AMPK. The AMPK-like kinase salt-inducible kinase (SIK) and class II histone deacetylases, along with expression of the HDAC target gene *Mef2c*, were also altered, supporting an impairment of LKB1-SIK-class II histone deacetylase signaling. Our results demonstrate that LKB1 may be involved in dystrophic progression, paving the way for future preclinical studies.

## Teaser

**Summary:** This study investigates the mechanical-metabolic uncoupling of dystrophic myofibers and the possible alteration of liver kinase B1, as a key player in modulating metabolism and epigenetic regulation of skeletal muscle.

## INTRODUCTION

The liver kinase B1 (LKB1), also known as serine/threonine protein kinase 11 (STK11) is a highly conserved protein across species, with emerging roles in various cellular processes, such as cellular polarity and adhesion, apoptosis, and control of the energetic metabolism (Shan et al., 2014). LKB1 activity is also important for skeletal muscle homeostasis, in particular for maturation and functions of stem cells with a positive control on myogenic differentiation, a crucial process for postnatal development and muscle repair after injury (Shan et al., 2017, 2014). In addition, mice with LKB1 deletion display skeletal muscle atrophy accompanied by inflammation, a reduction of mitochondrial content, and impaired muscle function (Chen et al., 2020). Intriguingly, these animals were exercise intolerant, with an *in vivo* impaired oxygen uptake and limited fatty acid oxidation; ectopic lipid accumulation has also been observed in both muscle precursors and mature muscles (Jeppesen et al., 2013).

LKB1 kinase activation occurs with the formation of a heterotrimeric complex with two accessory proteins, the pseudokinase STE20-related adaptor α (STRADα) and the scaffolding mouse protein 25 (MO25; also known as CAB39) (Zeqiraj et al., 2009). Although the exact subcellular localization of the complex remains unclear, especially in myofibers, a plausible hypothesis is that LKB1 localizes at the nuclear level and the binding with STRADα and MO25 induces the shuttling of LKB1 to the cytoplasm where the kinase activity is increased by tenfold (Boudeau, 2003).

One of the most important pathways regulated by LKB1 is the phosphorylation of adenosine monophosphate-activated protein kinase (AMPK), a ubiquitous master regulator of mitochondrial biogenesis and metabolic processes, which is physiologically activated in skeletal muscle under conditions of energetic stress, e.g. physical exercise and a high cytosolic AMP/ATP ratio (McGee and Hargreaves, 2010; Herzig and Shaw, 2018). Recent studies demonstrated that LKB1 also phosphorylates other AMPK-related kinases, including the salt-inducible kinases SIK1, -2 and -3 (Lizcano et al., 2004; Sun et al., 2020). Importantly, SIKs and AMPK are also involved in the modulation of epigenetic mechanisms by controlling class IIa histone deacetylase (HDAC) activity. In detail, HDAC4 and HDAC5, the main isoforms expressed in skeletal muscle, shuttle between nucleus and cytoplasm according to their phosphorylated state. In the non-phosphorylated state, HDACs have low affinity for cytoplasmic 14-3-3 phosphoserine-containing motif proteins and are then localized in the nucleus where they act as silencers of target genes (Wang et al., 2000). Conversely, phosphorylation promotes the nuclear export, cytosol sequestration and inactivation of the deacetylases (McGee and Hargreaves, 2010). SIKs and AMPK inactivate HDAC5 and HDAC4, by phosphorylation on serine-259 and -498 (Berdeaux et al., 2007; McGee and Hargreaves, 2010; Walkinshaw et al., 2013). The consequent inactivation of HDAC4 and HDAC5 allows the transcription of genes involved in oxidative and glucose metabolism, such as myocyte enhancer factor 2c (*MEF2C*), *GLUT-4* (*SLC2A4*) and peroxisome proliferator-activated receptor-γ co-activator 1 (*PGC-1α; PPARGC1A*), involved in mitochondrial biogenesis (McGee and Hargreaves, 2010).

Based on the above information, we questioned whether LKB1 could therefore be an important, yet unexplored, upstream player in progressive muscle-wasting related to the metabolic sufferance and intolerance to mechanical stress observed in Duchenne muscular dystrophy (DMD), as a consequence of the main primary defect, i.e. the absence of dystrophin (Duan et al., 2021; Gao and McNally, 2015). In fact, functional and metabolic adaptation appears to be faulty in dystrophic myofibers, as disclosed by the use of chronic exercise protocols in the C57BL/10ScSn-Dmd*^mdx^*/J (*mdx*) mouse, the most widely used model of DMD (Camerino et al., 2014; Capogrosso et al., 2017; Gamberi et al., 2018). Notably, exercised *mdx* mice display an insufficient AMPK phosphorylation, along with a defect in activation of PGC-1α and other players of mitochondrial oxidative metabolism; this can contribute to the reinforcement of inflammation and oxidative stress-related damaging signals (Capogrosso et al., 2017). A failure in proper AMPK signaling might contribute to inefficient inactivation of class II HDACs, reinforcing the epigenetic defects described for class I HDACs in dystrophic myofibers (Consalvi et al., 2011), a topic that deserves deep investigation in view of the high potential of HDAC inhibitors for DMD therapy (Bettica et al., 2016; clinical trial NCT02851797).

In this work, we evaluated whether an alteration of LKB1 upstream signaling might contribute to improper AMPK activation or SIK phosphorylation in DMD myofibers. In particular, we investigated the expression levels of the LKB1-MO25-STRADα complex and the phosphorylation state of key downstream metabolic and epigenetic players, AMPK, SIK2 and class II HDACs, in dystrophic animal models. Other than classic *mdx* mice, the more recent D2.B10-Dmd*^mdx^*/ J (D2 *mdx*) mouse model was used. The D2 *mdx* model is believed to have a more severe phenotype than that of classic *mdx* mice, owing to the presence of polymorphisms acting as disease modifiers (Coley et al., 2016). These can account for a more rapid deterioration of muscle and cardiac function and an exacerbation of inflammatory and pro-fibrotic events, along with defects in energy metabolism and mitochondrial function, making this a model of interest for our working hypothesis (Ramos et al., 2020). In addition, we also investigated the cross-talk between LKB1 and AMPK in *mdx* mice after physiological stimulation (i.e. exercise) and pharmacological stimulation through metformin, an indirect AMPK stimulator with a well-recognized efficacy and safety profile, recently tested in people with DMD in view of its anabolic potential (clinical trial NCT02516085) (Camerino et al., 2014; Mantuano et al., 2018). The overall goal of this study is to gain more insight into the early signaling processes underlying the impaired metabolic adaptation resulting from dystrophin absence in myofibers, and possibly identify targets for developing innovative therapeutic approaches.

## RESULTS

### Basic characteristics of dystrophic mice: body mass, GC muscle weight, and CK plasma levels

Body mass, as well as gastrocnemius (GC) weight and creatine kinase (CK) plasma levels, of *mdx* and D2 *mdx* mice and their respective wild-type (WT) counterparts are reported in Table 1. The body mass of WT and D2 WT mice at sacrifice was similar, whereas *mdx* and D2 *mdx* mice exhibited an opposite trend, with a statistically significant weight gain for *mdx* mice and a significant reduction of body mass for D2 *mdx* with respect to the related WT groups. Analysis of GC muscle weight revealed a similar result. In fact, *mdx* mice showed an increase of GC muscle mass compared with WT, in accordance with the muscle hypertrophy widely described for this specific dystrophic murine model (Duddy et al., 2015; Faber et al., 2014). Conversely, D2 *mdx* mice exhibited a severe reduction of GC weight compared with D2 WT mice, although the latter were characterized per se by a diminished muscle mass with respect to WT. The reduced muscle mass in D2 *mdx* GC was also detectable when individual values normalized to mouse body mass were calculated.

**Table 1.**
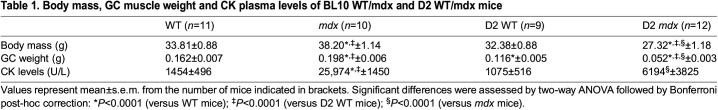
Body mass, GC muscle weight and CK plasma levels of BL10 WT/mdx and D2 WT/mdx mice

CK plasma concentration, an index of muscle damage, was, as expected, markedly increased (>1000%) in *mdx* mice compared with WT mice (Hathout et al., 2014). The CK increase was also detected in D2 *mdx* mice compared with D2 WT, although it was milder than that observed in classical *mdx* mice, likely as a result of the reduced total muscle tissue.

### Analysis of LKB1-MO25-STRADα complex integrity in dystrophic settings

#### LKB1 expression and localization in GC muscles

**Fig. 1.**
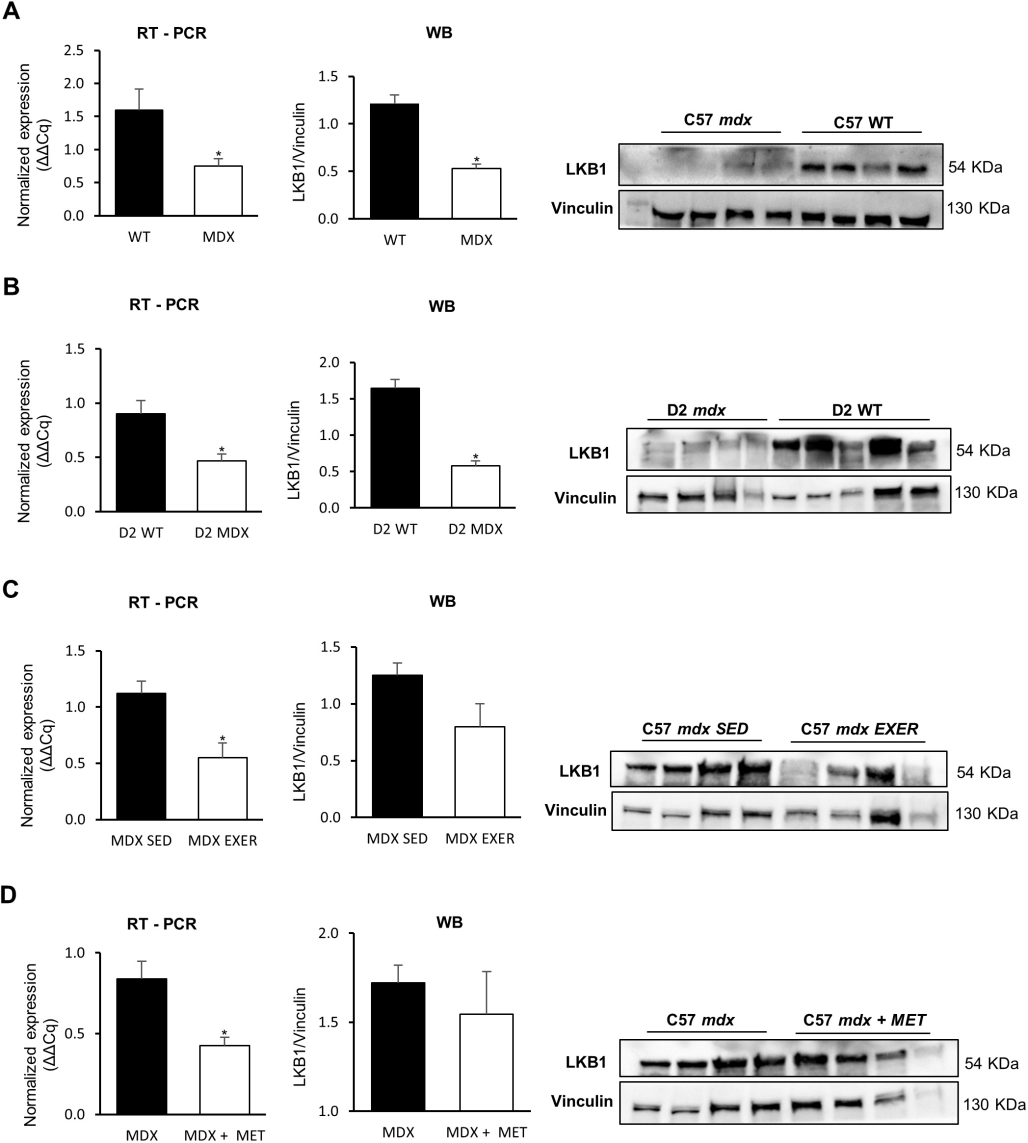
**Gene and protein expression of LKB1 in GC muscles of all experimental groups.** (A-D) RT-PCR and western blot (WB) analyses of LKB1 expression in the following mice: (A) C57BL/10 *mdx* mice (*n*=10) versus C57BL/10 WT mice (*n*=11) (*P<0.036); (B) D2 *mdx* mice (*n*=12) versus D2 WT mice (*n*=9) (*P<0.0192); (C) C57BL/10 *mdx* exercised (EXER) mice (*n*=4) versus C57BL/10 *mdx* sedentary (SED) mice (*n*=4) (*P=0.0153); (D) C57BL/10 *mdx* mice treated with metformin (MET) (*n*=4) versus C57BL/10 *mdx* mice (*n*=4) (*P=0.015). Blots were loaded by an operator unaware of the experimental groupings of samples. In A, the most representative blot of the three western blot experiments performed is shown because of the large number of samples belonging to Group 1. Data are mean+s.e.m. Significant differences were assessed by unpaired Student's *t*-test.

Fig. 1 shows both *Lkb1* gene expression and LKB1 protein levels measured in all experimental groups. Downregulation of the *Lkb1* gene and a parallel reduction of protein levels by western blot analysis were found in both *mdx* and D2 *mdx* mice compared with their respective WT controls (Fig. 1A,B).

Interestingly, *mdx* mice undergoing a chronic (20 week) exercise protocol, exhibited a further remarkable reduction of *Lkb1* gene expression level compared with *mdx* mice. Furthermore, a trend towards reduction, although not significant, was also observed at the protein level (Fig. 1C). Surprisingly, *Lkb1* transcript levels were also halved in *mdx* mice treated with metformin for 20 weeks compared with untreated mice, although this result was not confirmed at the protein level (Fig. 1D).

**Fig. 2.**
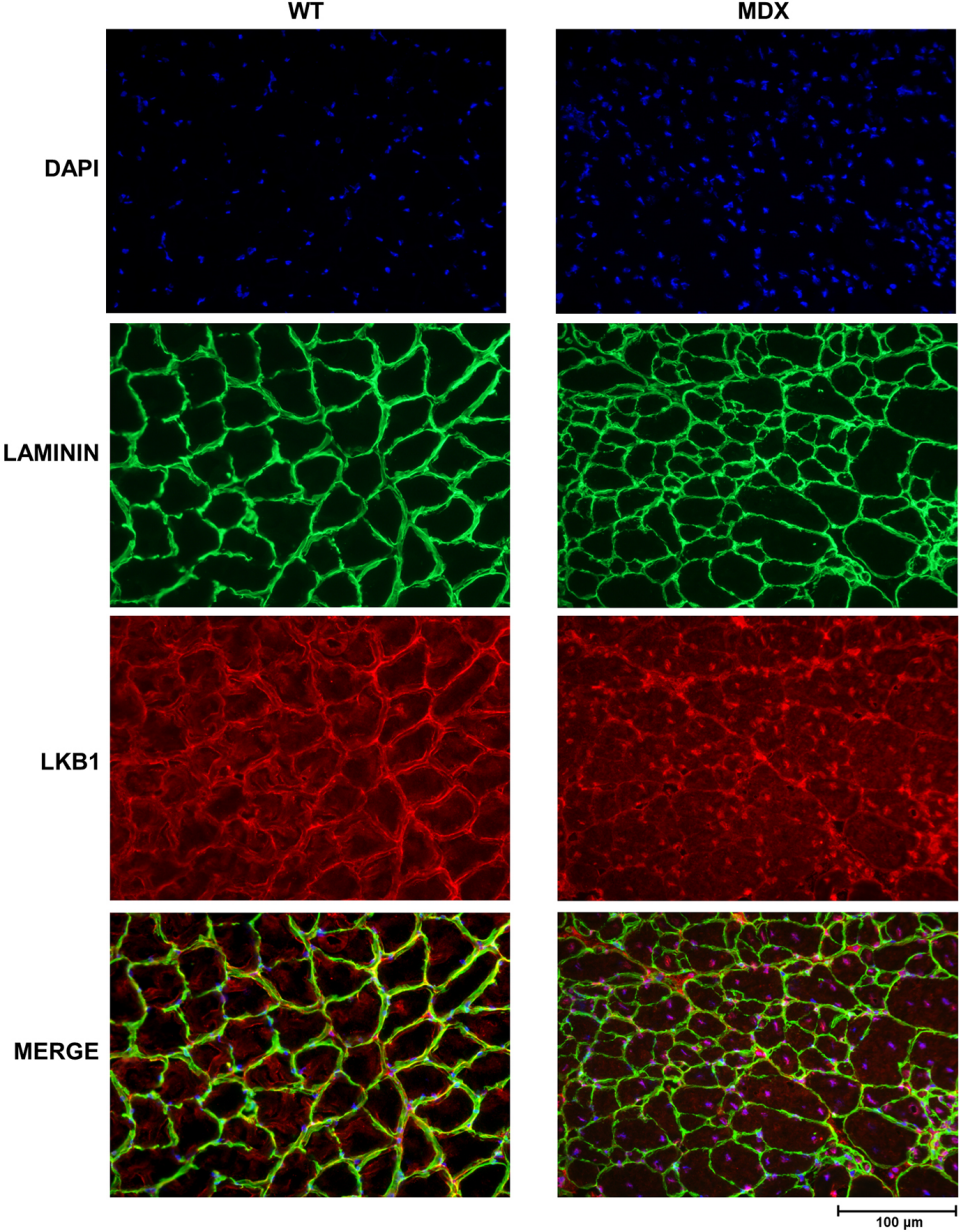
**Immunofluorescence imaging for LKB1.** GC muscle sections of 6-month-old BL10 *mdx* and WT mice were stained with antibodies against laminin as a sarcolemmal marker (green) and LKB1 (red). Nuclei were stained with DAPI (blue). RGB merged images of the three channels are shown at the bottom.

Immunofluorescence analysis (Fig. 2), performed on GC muscle tissues, revealed that LKB1 was widely distributed and localized at the sarcolemmal level in WT mice. Conversely, tissue sections of *mdx* mice displayed a reduced immunofluorescence signal for LKB1 that markedly colocalized with DAPI at the nuclear level.

#### Protein levels of MO25 and STRADα

**Fig. 3.**
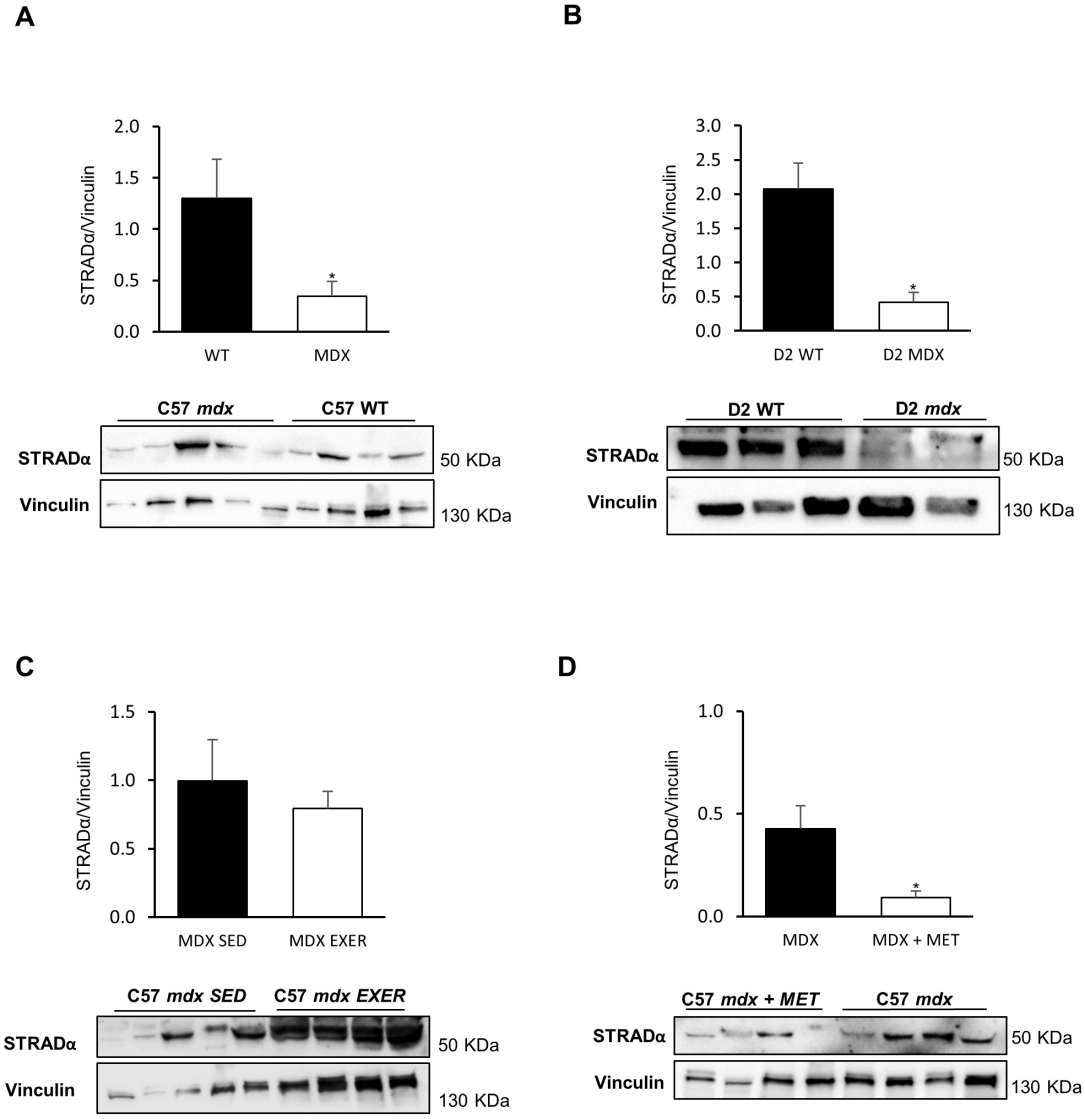
**STRADα protein expression in GC muscles of different animal models and in various experimental conditions.** Western blot analyses of STRADα expression in the following mice: (A) C57BL/10 *mdx* mice (*n*=4) versus C57BL/10 WT mice (*n*=3) (*P=0.0444); (B) D2 *mdx* mice (*n*=3) versus D2 WT mice (*n*=3) (*P=0.0402); (C) C57BL/10 *mdx* exercised (EXER) mice (*n*=3) versus C57BL/10 *mdx* sedentary (SED) mice (*n*=5). (D) C57BL/10 *mdx* mice (*n*=4) versus C57BL/10 *mdx* mice treated with metformin (MET) (*n*=3) (*P=0.0191). Blots were loaded by an operator unaware of the experimental groupings of samples. Data are mean+s.e.m. Significant differences were assessed by unpaired Student's *t*-test.

**Fig. 4.**
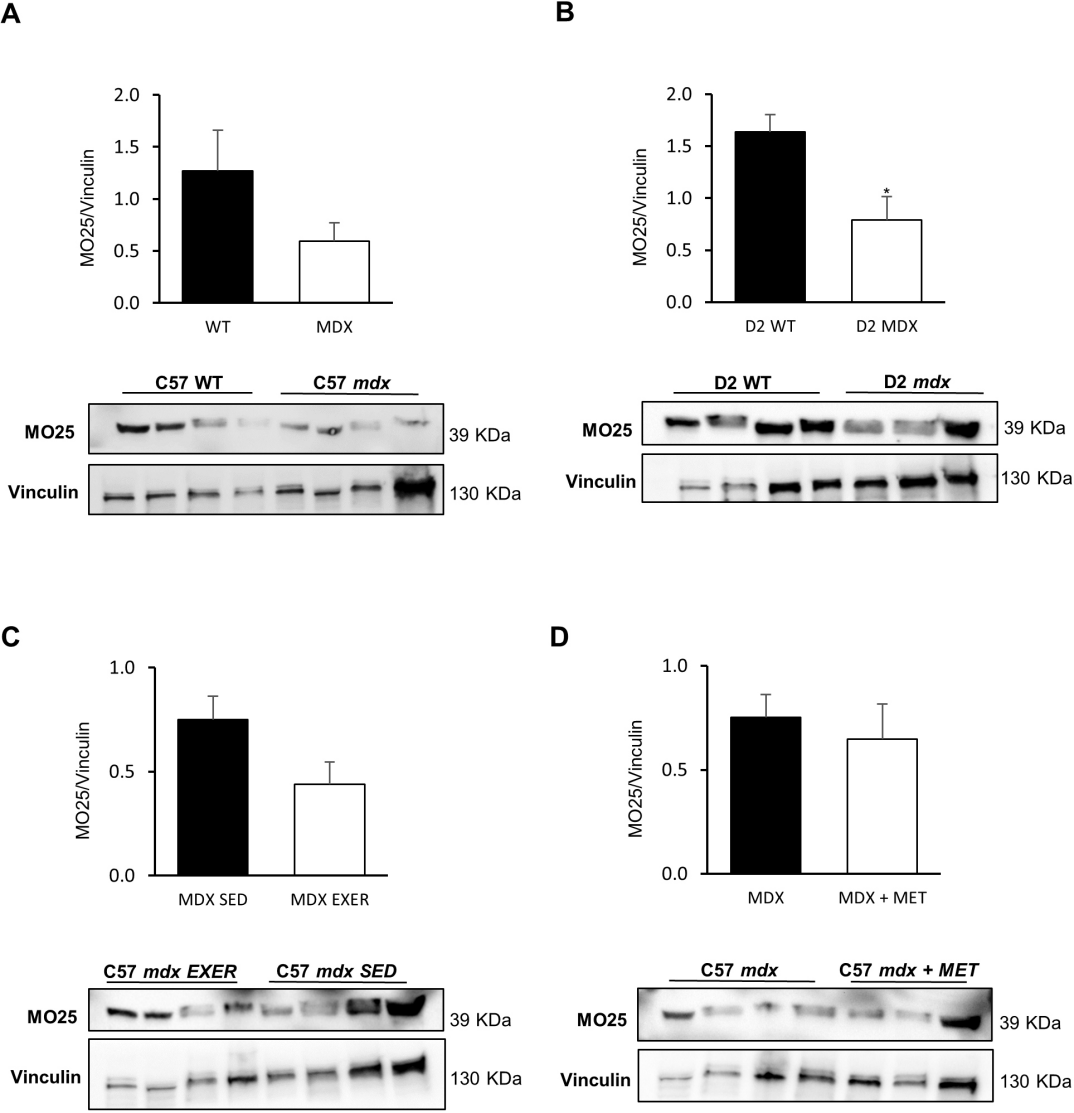
**MO25 protein expression in GC muscles of different animal models and in various experimental conditions.** Western blot analyses of MO25 expression in the following mice: (A) C57BL/10 *mdx* mice (*n*=4) versus C57BL/10 WT mice (*n*=4); (B) D2 *mdx* mice (*n*=3) versus D2 WT mice (*n*=3) (*P=0.0267); (C) C57BL/10 *mdx* exercised (EXER) mice (*n*=4) versus C57BL/10 *mdx* sedentary (SED) mice (*n*=4); (D) C57BL/10 *mdx* mice (*n*=4) versus C57BL/10 *mdx* mice treated with metformin (MET) (*n*=4). Blots were loaded by an operator unaware of the experimental groupings of samples. Data are mean±s.e.m. Significant differences were assessed by unpaired Student's *t*-test. Data are mean+s.e.m. Significant differences were assessed by unpaired Student's *t*-test.

The results of protein analysis of MO25 and STRADα, the two accessory proteins forming the heterotrimeric complex with LKB1, are shown in Figs 3 and 4, for all experimental groups.

Both proteins, and in particular STRADα, were markedly reduced in *mdx* mice of each strain (BL10 and D2) compared with the corresponding control groups, following the pattern observed for LKB1 (Figs 3A,B, 4A,B). The exercise protocol induced a trend toward a further reduction in the level of both proteins, which was again more evident for STRADα (Figs 3C, 4C). Metformin treatment (Figs 3D, 4D) did not change MO25 level; however, the STRADα level was further reduced with respect to untreated *mdx* mice.

### Protein and gene expression analysis of LKB1 downstream targets

**Fig. 5.**
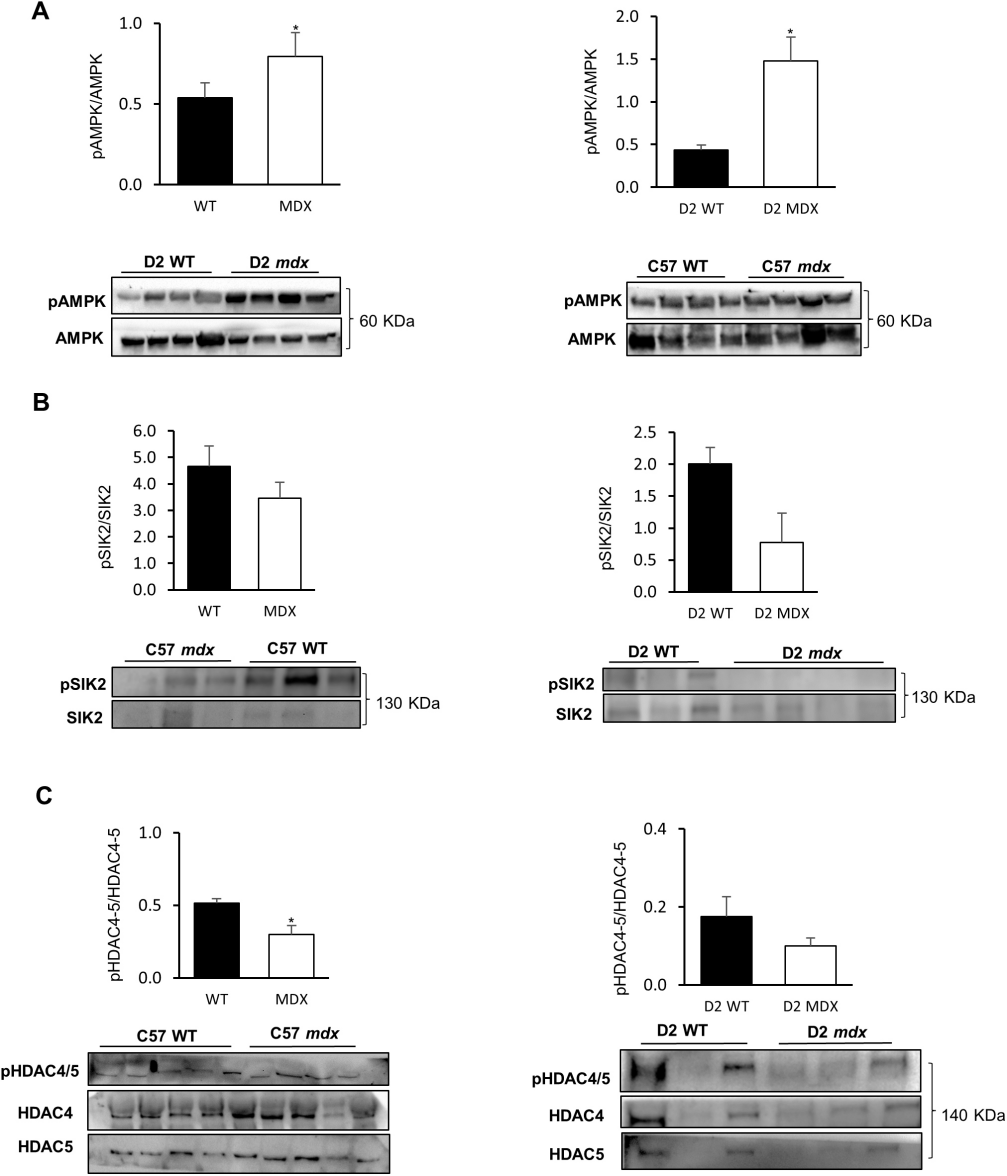
**pAMPK/AMPK, pSIK2/SIK2 and pHDAC4-5/(HDAC4+HDAC5) ratio in GC muscles of different animal models.** (A) Western blot analysis of pAMPK/total AMPK in C57BL/10 *mdx* mice (*n*=6) versus C57BL/10 WT mice (*n*=9) (*P=0.044), and in D2 *mdx* mice (*n*=8) versus D2 WT mice (*n*=6) (*P=0.0088). (B) Western blot analysis of pSIK2/total SIK2 in C57BL/10 *mdx* mice (*n*=7) versus C57BL/10 WT mice (*n*=6), and in D2 *mdx* mice (*n*=6) versus D2 WT mice (*n*=6). (C) Western blot analysis of C57BL/10 *mdx* mice (*n*=4) versus C57BL/10 WT mice (*n*=4) (*P=0.0002) and D2 *mdx* mice (*n*=6) versus D2 WT mice (*n*=6) (not significant). Blots were loaded by an operator unaware of the experimental groupings of samples. Data are mean+s.e.m. Significant differences were assessed by unpaired Student's *t*-test.

Given that a reduced level of LKB1 and of its co-activators could account for a possible abnormal modulation of downstream metabolic players, we measured the phosphorylated state of LKB1 targets. We started by measuring the phosphorylated (activated) pAMPK/total AMPK ratio in basal conditions (Fig. 5A). In our previous studies, we found a slight increase in basal phosphorylation of AMPK, but neither exercise nor metformin treatment improved the pAMPK/AMPK ratio in *mdx* mice (Capogrosso et al., 2017; Mantuano et al., 2018). In the present study, we observed again an increased pAMPK/AMPK ratio in *mdx* mice compared with age-matched controls; in addition, we found, for the first time, a significant increase of pAMPK/AMPK ratio in D2 *mdx* compared with D2 WT mice.

Then, we investigated the phosphorylation ratio of SIK2. Interestingly, the pSIK2/SIK2 ratio was reduced in *mdx* mice of both strains, being more evident in the D2 background (Fig. 5B), disclosing for the first time an impairment of SIK signaling in a dystrophic setting.

In order to investigate the correlation between the altered SIK2/AMPK activation and the extent of class II HDAC phosphorylation, we analyzed the ratio between the levels of phosphorylated (and inactive) forms of HDAC4 and HDAC5 proteins compared with total HDAC4 and HDAC5, for both strains (Fig. 5C). Interestingly, *mdx* mice exhibited a significantly reduced pHDAC4-5/(HDAC4+HDAC5) ratio compared with WT mice. A similar trend was observed in D2 *mdx* mice, although the difference compared with D2 WT was not statistically significant.

**Fig. 6.**
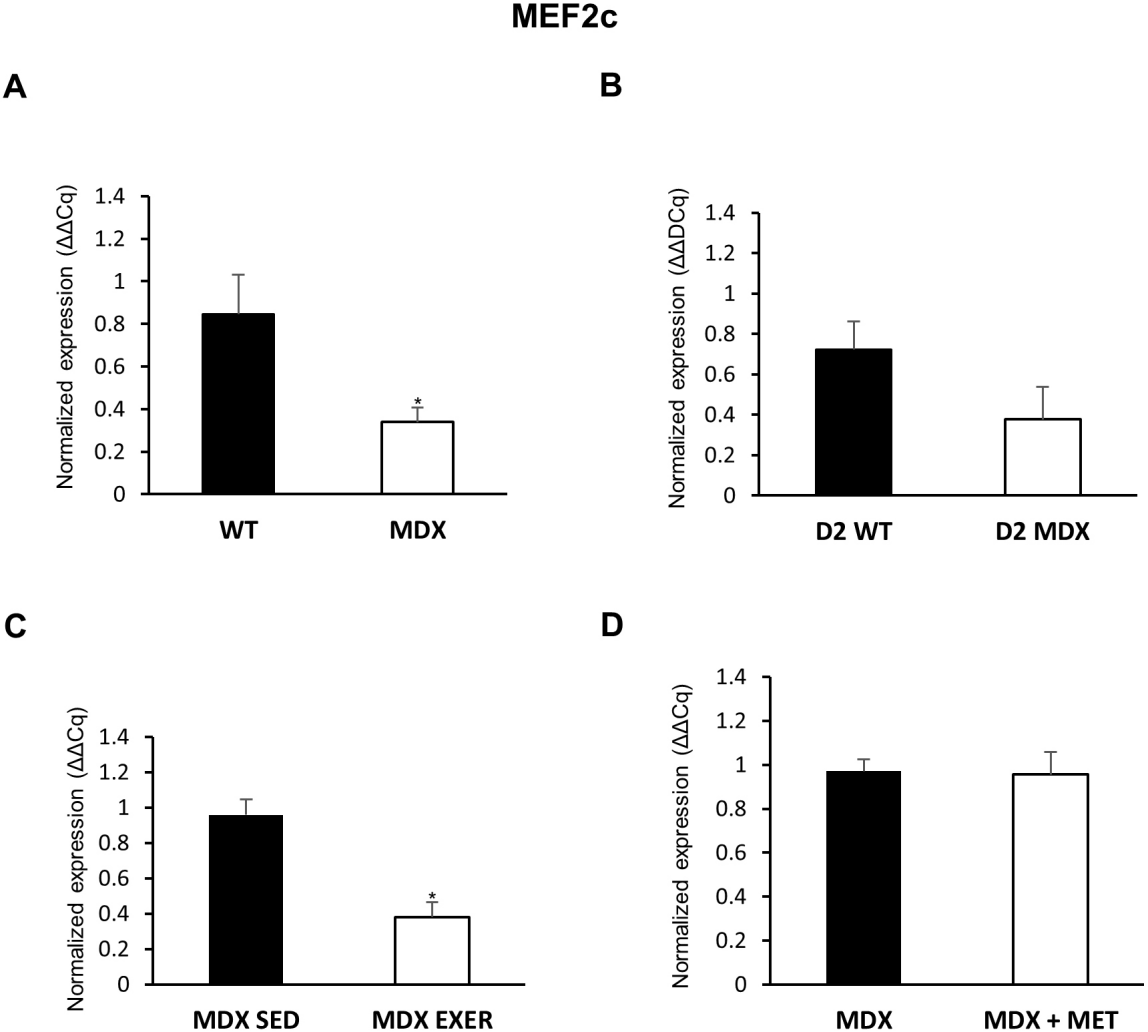
**Expression of *Mef2c* gene in GC muscles of all experimental groups.** RT-PCR analysis of *Mef2c* expression in the following mice: (A) C57BL/10 *mdx* mice (*n*=4) versus C57BL/10 WT mice (*n*=3) (*P=0.0328); (B) D2 *mdx* mice (*n*=4) versus D2 WT mice (*n*=4); (C) C57BL/10 *mdx* exercised (EXER) mice (*n*=4) versus C57BL/10 *mdx* sedentary (SED) mice (*n*=4) (*P=0.003); (D) C57BL/10 *mdx* mice (*n*=4) versus C57BL/10 *mdx* mice treated with metformin (MET) (*n*=4). Data are mean±s.e.m. Significant differences were assessed by unpaired Student's *t*-test.

The gene expression levels of *Mef2c*, a primary target silenced by HDAC4 and HDAC5, were significantly reduced in *mdx* GC muscle, and a clear trend toward reduction was found in D2 *mdx* mice (Fig. 6), in line with the low phosphorylation levels of HDAC4 and HDAC5 previously described (Fig. 5). Exercised *mdx* mice showed a marked decrease in *Mef2c* expression compared with *mdx* mice, in line with a potential faulty adaptation to exercise (Camerino et al., 2014). Finally, metformin administration did not affect the expression levels of *Mef2c*.

### Analysis of LKB1 expression and localization in healthy and dystrophic muscle precursors

**Fig. 7.**
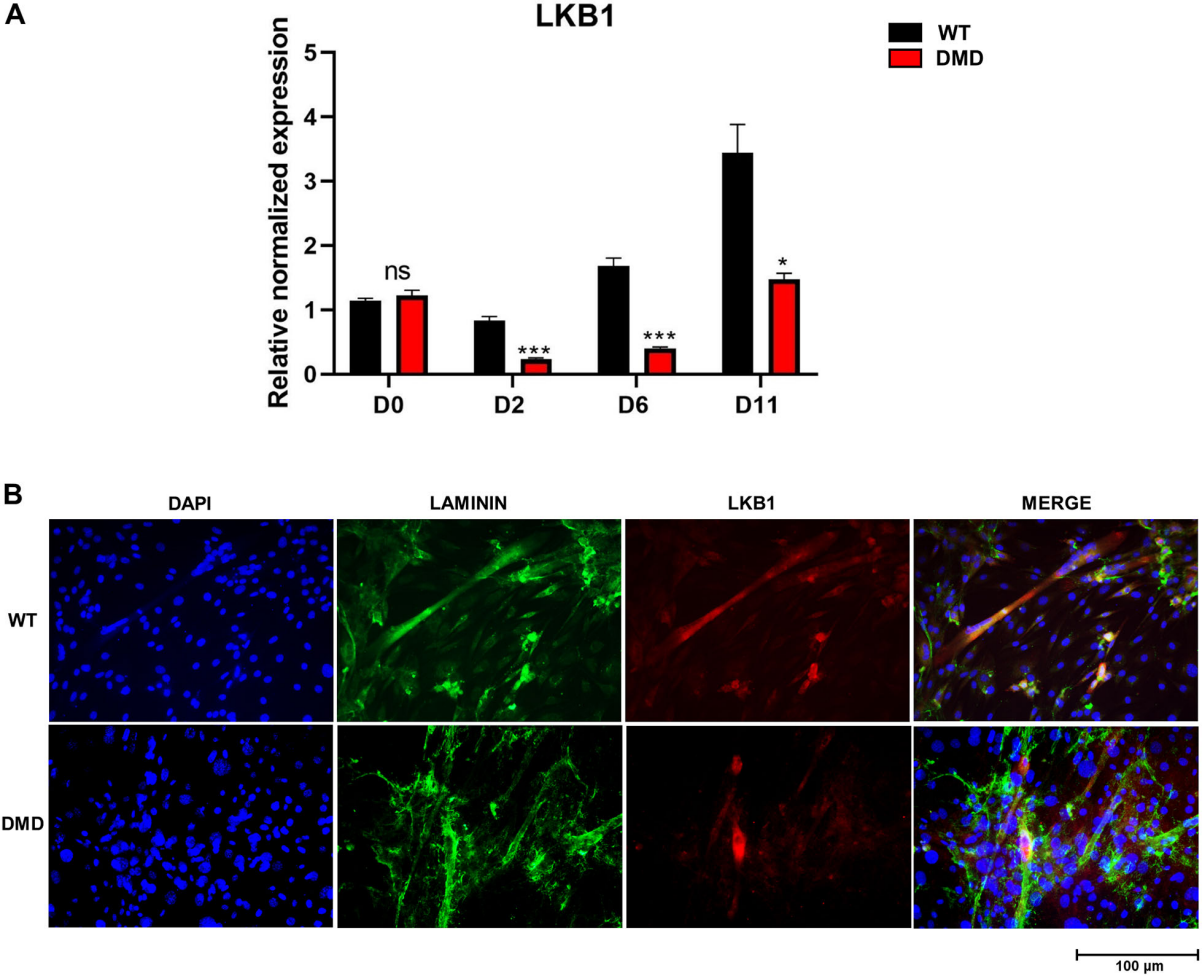
**Gene expression and localization of LKB1 in WT and dystrophic murine muscle cells.** (A) *Lkb1* expression in 2B4 (WT) and SF1 (DMD) myoblasts (D0), and myocytes (D2-D11). Significant differences were assessed by unpaired Student's *t*-test (*P≤0.05; ***P≤0.001). ns, not significant. (B) Immunofluorescence imaging for LKB1. D11 2B4 and SF1 cells were stained with antibodies against laminin as a sarcolemmal marker (green) and LKB1 (red). Nuclei were stained with DAPI (blue). RGB merged images of the three channels are shown on the right.

In order to evaluate whether the alteration in LKB1 expression is a downstream consequence of the complex cascade of pathology events occurring in *mdx* mouse muscles, such as oxidative stress and inflammation, or rather directly related to the primary dystrophin deficiency, we assessed *Lkb1* expression in muscle precursors throughout *in vitro* myogenesis, by RT-PCR analyses using healthy 2B4 (WT) and dystrophic SF1 (DMD) cell lines, as myoblasts or at various time points of differentiation [day (D) 2, D6 and D11] (Fig. 7A). Notably, both WT and dystrophic myoblasts showed similar levels of LKB1. This latter increased in a time-dependent manner in WT myocytes during differentiation, whereas a significant downregulation of *Lkb1* (of about 70%) was detected at all time points in differentiating dystrophic cells. Moreover, to assess the LKB1 localization at this stage, we performed immunofluorescence experiments on WT and DMD cells at D11 of differentiation (Fig. 7B). Notably, we observed a widespread distribution of the kinase in healthy cells, mainly present at the membrane level, whereas in dystrophic cells the immunofluorescence signal was weaker and mainly localized at the nuclear level.

## DISCUSSION

The understanding of disease-related factors governing the impaired cross-talk between metabolism and epigenetic mechanisms in Duchenne muscular dystrophy is still in its infancy. To shed light on this aspect, in the present work we specifically focused on some major metabolic and epigenetic players in two main dystrophic murine models, *mdx* and D2 *mdx* mice. Their different phenotypes, also evidenced by macroscopic features such as body and muscle mass and CK level, highlight common traits versus secondary changes, allowing exploration of the alterations underlying the primary pathology (Van Putten et al., 2019; Vohra et al., 2017).

Our main focus was to deepen our knowledge of the potential role of LKB1 as upstream player in the sufferance of metabolic hub and in epigenetic modulation with particular attention to AMPK and class II HDAC signaling. The main finding of our study was a marked reduction of LKB1 expression in *mdx* mice of 6 months of age, i.e. during the phase of stable chronic damage, paralleled by low levels of MO25 and STRADα synthesis, the two proteins involved in forming and activating the ternary complex that shuttles from the nucleus into cytoplasm. Accordingly, our immunofluorescence staining showed that *mdx* GC muscle sections had a faint LKB1 signal, mainly present at in the nucleus, in contrast with the expected localization at the sarcolemma observed in WT muscle sections (Dogliotti et al., 2017). Notably, a similar extent of LKB1 downregulation and low levels of MO25 and STRADα were found in the D2 *mdx* mouse model, supporting the overall impairment of the heterotrimeric complex as a key feature of muscular dystrophy. Importantly, downregulation of MO25 and STRADα might be involved not only in the mislocalization of LKB1, but also in the impairment of its expression levels, perhaps through the interaction with specific inhibitory miRNAs (Chen et al., 2012), a hypothesis that deserves dedicated efforts.

Importantly, we also found that LKB1 was markedly reduced in dystrophic myocytes during differentiation, whereas in WT cells we observed a time-dependent increase. This finding not only corroborates that LKB1 is an early key player of myogenic program, but highlights that its impairment in dystrophic settings is directly related to the intrinsic genetic alteration, and not a secondary consequence of disease progression.

The upregulation of LKB1 gene expression in healthy WT cells during myogenic differentiation ties in well with the described role of this kinase in the cytoskeletal changes required for myotube formation, and its persistent impairment could, in part, account for the inefficient myogenic program of dystrophic muscle over time (Mian et al., 2012).

**Fig. 8.**
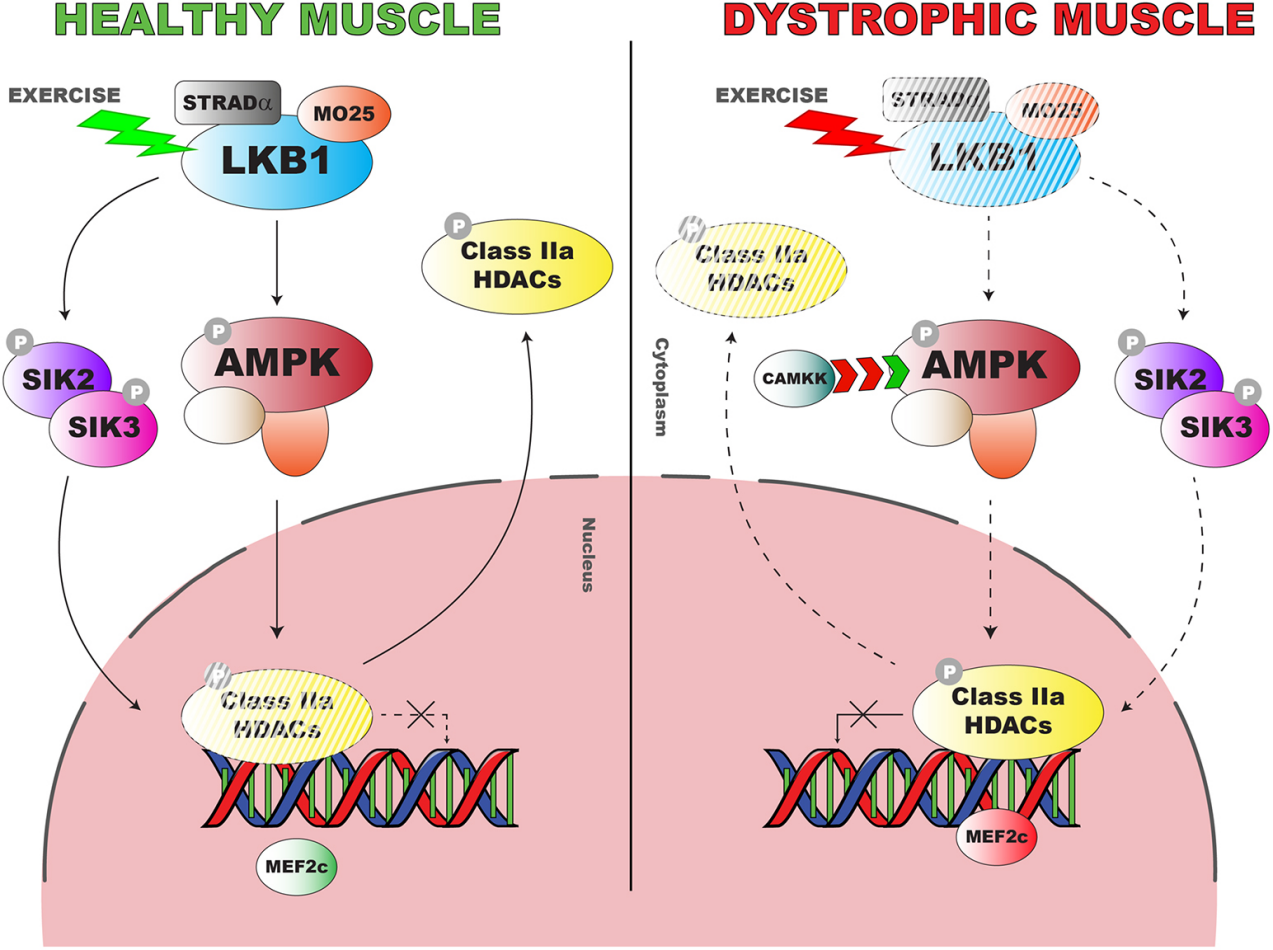
**Schematic summarizing the pathways involved in the LKB1-AMPK-HDAC axis in healthy and dystrophic muscle.** In healthy muscle, LKB1 is activated in response to exercise, in turn stimulating the metabolic remodeling mediated by the key modulator AMPK. AMPK turns off the class II HDACs that are also inhibited via SIK phosphorylation by LKB1. In dystrophic muscle, this refined mechanism of regulation is altered owing to LKB1 impairment. This allows class II HDACs to carry on their epigenetic silencer action, thus inhibiting the expression of genes involved in oxidative metabolism (*Mef2c*).

AMPK is known to be a primary target of LKB1 (Hawley et al., 2003). However, dystrophic muscles are characterized by a significant basal increase of AMPK phosphorylation (Capogrosso et al., 2017; Mantuano et al., 2018), which we confirmed herein also in D2 *mdx* mice. AMPK activation is strictly related to low-energy conditions, leading to reduction of anabolism and promotion of catabolic processes. Nonetheless, chronic activation in basal conditions may have deleterious effects on muscle homeostasis, inhibiting muscle growth and stimulating pro-atrophic pathways (Thomson, 2018). The basal AMPK overactivation observed in *mdx* and D2 *mdx* mice in the presence of an impairment of LKB1 can be reconciled by the existence of additional mechanisms responsible for AMPK phosphorylation, possibly de-regulated in dystrophic conditions. In particular, Ca^2+^/calmodulin-dependent protein kinase 2 (CAMKK2), which is known to be overexpressed and overactive in DMD in relation to alteration in calcium homeostasis (Ye et al., 2016), can phosphorylate AMPK at the same site targeted by LKB1 (Tojkander et al., 2018) and might represent a likely pathway for basal AMPK phosphorylation (Fig. 8).

In parallel, AMPK is not the unique target of LKB1. In fact, analysis of SIK2 phosphorylation ratio revealed, for the first time, reduced activation of the kinase in GC muscle of *mdx* mice of both strains compared with their respective WT, providing a feasible parallel mechanism linking LKB1 reduction with epigenetic alteration, via the described role of SIKs on phosphorylation of class II HDACs (Sun et al., 2020). In fact, despite the increased activation of AMPK in *mdx* mice, we observed inefficient phosphorylation of HDAC4 and HDAC5, which could maintain their role as epigenetic silencers, turning off the expression of target genes, such as *Mef2c*. We cannot rule out that this effect might also depend on a mislocalization of pAMPK/AMPK with a consequent de-regulated target engagement. This hypothesis requires further investigation. A similar pattern was detected in D2 *mdx* mice, although the pHDAC4-5/ (HDAC4+HDAC5) ratio was reduced in D2 WT mice compared with BL10 WT.

Interestingly, a chronic protocol of exercise, known to worsen the inflammatory and the dysmetabolic condition in the *mdx* mouse model (Camerino et al., 2014), further impaired the level of *Lkb1* transcript and, to a lesser extent, of the LKB1 protein level, likely in relation to the turnover and half-life of the protein. Moreover, the translation of MO25 and STRADα was further reduced. This suggests that the alteration of the heterotrimeric complex is related to aberrant mechanical transduction, providing a clue for the basis of the lack of AMPK activation observed in exercised *mdx* mice (Capogrosso et al., 2017; Mantuano et al., 2018). A possible explanation for the opposite patterns of AMPK activation observed in basal and energetic stress conditions in *mdx* mice may lie in the different signals driving phosphorylation of the kinase in dystrophic myofibers. We suggest that in basal conditions the chronic high intracellular Ca^2+^ concentrations stimulate CAMKK2, thereby promoting a basal compensatory AMPK activation. In contrast, in conditions of energetic stress caused by physical exercise, impaired LKB1 signaling fails to efficiently transduce mechanical-metabolic coupling, leading to an insufficient stimulation of AMPK in dystrophic myofibers. Moreover, during exercise AMPK activation is also regulated by the fluctuation of energy-related metabolites, such as phospho-creatine, ATP and, most importantly, AMP, which allosterically facilitate AMPK phosphorylation by LKB1 (Hancock et al., 2006). Interestingly, exercised *mdx* mice showed a severe downregulation of adenylate kinase 1 (AK1) (Gamberi et al., 2018), the main enzyme that catalyzes AMP synthesis during muscle contraction, suggesting an overall failure of AMPK activation pathways in relation to exercise.

A similar mechanism could account for the rather mild increase in pAMPK/AMPK ratio observed in metformin-treated *mdx* mice (Mantuano et al., 2018), given that this drug classically works by activating AMPK via complex I inhibition in mitochondria and increasing the AMP level. However, we unexpectedly found that dystrophic mice treated with metformin showed a further downregulation of *Lkb1* gene expression accompanied by a reduced level of STRADα protein. To date, very little is known about upstream regulation of *Lkb1*, and the specific reason for the apparent negative effect of metformin administration on its expression remains unclear. Again, a role for epigenetic control by miRNAs cannot be ruled out, which will require further investigation.

### Conclusions

Our study provides a new insight into the role of LKB1 as a possible player in the defective metabolic signaling observed in dystrophic conditions. Our data show for the first time a global impairment of LKB1-STRADα-MO25 complex stability in both dystrophic mouse models and cell lines, although the upstream mechanism underlying this condition remains unclear. This event could have a causal role in the subsequent failure of correct cellular signal transmission, accounting for the aberrant metabolic response in dystrophic myofibers. Our results pave the way for future research that could continue to explore the contribution of altered epigenetic background and the actual role of the LKB1-AMPK-HDACIIa axis in the mechanical-metabolic uncoupling of dystrophic muscle. The potential trigger of an auto-reinforcing loop should be considered, in view of the ability of the HDAC inhibitor givinostat to rescue the decrease in PGC-1α expression in dystrophic *mdx* muscles (Giovarelli et al., 2021). Further analyses are also required to explore the involvement of LKB1 phosphorylation, the most important post-translational modification, in the regulation of LKB1 activity and its downstream mechanisms.

In this scenario, drugs able to restore proper functionality of LKB1 could be of interest, considering its upstream and early intrinsic alteration in dystrophic settings. To date, no pharmaceutical compounds have been identified that are able to increase LKB1 activity. However, potential epigenetic modulation of LKB1 or massive degradation of the kinase transcript exerted by specific miRNAs should be explored as potential opportunities for pharmacological modulation. Overall, our results pave the way to an innovative research line, which could be useful for the identification of drug targets and to improve pharmacological interventions in translational research in DMD.

## MATERIALS AND METHODS

All experiments were conducted in compliance with the Italian law for Guidelines for Care and Use of Laboratory Animals (D.L. 116/92), and European Directive (2010/63/UE). Most of the experimental procedures respected the standard operating procedures for pre-clinical tests in *mdx* mice available on the TREAT NMD website (http://www.treat-nmd.eu/research/preclinical/dmd-sops/).

### Study design

Experiments were performed on GC muscle samples from male *mdx* mice belonging to four experimental groups previously used for other studies (protocol number of Italian Ministry of Health approval: DGSAF0024012). In detail, the following experimental groups were used: Group 1: 6-month-old *mdx* mice (C57BL/10ScSn-Dmd*^mdx^*/J) (*n*=10) and age-matched male WT mice (C57BL/10 WT) (*n*=10); Group 2: 6- to 10-month-old D2.B10-Dmd*^mdx^*/J (D2 *mdx*, *n*=12) and related WT DBA/2J (D2 WT, *n*=10); Group 3: 6-month-old exercised *mdx* (*mdx* exer) mice (*n*=4); Group 4: 6-month-old *mdx* mice treated with metformin (*mdx*+met, *n*=4). For Group 3, 4-week-old *mdx* mice underwent a standard training protocol on a horizontal treadmill (Columbus Instruments; at 12 m/min, twice a week) (Grounds et al., 2008) for 20 weeks. For Group 4, the dose of metformin hydrochloride (1-tdimethylbiguanide hydrochloride, Sigma-Aldrich) was 200 mg/kg/day, formulated in filtered tap water and administered orally to 4-week-old *mdx* mice for 20 weeks (Mantuano et al., 2018). *mdx* mice from Group 1 were used as sedentary/untreated control animals for Groups 3 and 4. At sacrifice, body mass (g) of all mice belonging to Groups 1 and 2 was assessed. After isolation, both GC muscles were weighed and snap-frozen in liquid nitrogen and stored at −80°C until further processing for gene and protein expression analyses.

### Spectrophotometric determination of CK plasma levels

The enzymatic activity of CK in plasma samples of Groups 1 and 2 (in U/l) was determined using specific commercially available diagnostic kits (CK NAC LR, SGM Italia) by spectrophotometer determination (Ultrospec 2100 Pro UV/Visible, Amersham Biosciences) set to a wavelength of 340 nm at 37°C, according to the manufacturer's instructions (Capogrosso et al., 2017; Mantuano et al., 2021; Sanarica et al., 2019).

### Cell cultures

An immortalized mouse satellite cell-derived cell line (H2K-2B4, WT) and its dystrophic counterpart (H2K-SF1, DMD) (Muses et al., 2011) were cultured in proliferative (myoblasts) and differentiation medium (myocytes/myotubes), respectively. Myoblasts were cultured in growth medium composed as follows: DMEM high glucose with sodium pyruvate (Immunological Sciences), 20% (v/v) heat-inactivated fetal bovine serum South America (Immunological Sciences), 2% (v/v) chicken embryo extract (Thermo Fisher Scientific), 4 mM L-glutamine (Immunological Sciences) and 1% (v/v) penicillin/streptomycin (Immunological Sciences). Wells were coated with 0.1 mg/ml of Cultrex^®^ 3-D Culture Matrix RGF Basement Membrane Extract or Matrigel (Bio-Techne^®^), to encourage cell adhesion and incubated for 40 min at 33°C before removing any Matrigel excess. Each cell line contains a thermolabile t-antigen protein that maintains cells in the proliferative state. In order to initiate its expression, we added 20 U/ml of interferon gamma (γ-IFN; Sigma-Aldrich^®^) to the growth medium and we cultured 2B4/SF1 cells at 33°C with 5% CO_2_.

To trigger terminal differentiation, we cultured 2B4 and SF1 cells in differentiation medium, composed of DMEM, 5% (v/v) horse serum (Sigma-Aldrich^®^), 4 mM L-glutamine and 1% penicillin/streptomycin (Immunological sciences). Plates were coated with Matrigel as described above. Cells were cultured at 37°C with 5% CO_2_ in the absence of γ-IFN. We differentiated cell lines for 2-6 and 11 days, changing half of the differentiation medium every 2 days.

### Isolation of total RNA, reverse transcription, and real-time PCR

For each sample, total RNA was isolated from frozen right GC muscles with TRIzol^®^ reagent (15596026 and 15596018, Invitrogen) and quantified by spectrophotometry (ND-1000 NanoDrop, Thermo Fisher Scientific). To perform reverse transcription, the Advantage^®^ RT-for-PCR Kit (Clontech – Takara Bio) was used. In brief, 100 ng of total RNA was diluted in 12.5 µl of DEPC-treated H_2_O and was added with 1 µl of oligo(dT)_18_ primers. Then, the sample was incubated at 70°C for 2 min. Afterward, 4 µl of 5× Reaction Buffer, 1 µl of dNTP Mix, 0.5 µl of Recombinant RNase Inhibitor and 1 µl of MMLV Reverse Transcriptase were added and incubated at 42°C for 1 h, followed by 5 min at 94°C.

qRT-PCR was performed using the Applied Biosystems StepOnePlus™ Instrument (Applied Biosystems). Each reaction, carried out in duplicate, consisted in 1.5 ng of cDNA added with 5 µl of PowerUp SYBR Green PCR Master Mix (Applied Biosystems), 0.5 µl of primers and 6 µl of nuclease-free water for a final volume of 10 μl. All qPCR performed using SYBR Green was conducted at 50°C for 2 min, 95°C for 2 min, and then 40 cycles of 95°C for 15 s and 60°C for 1 min. The average mRNA expression of target genes was normalized to the mean of two housekeeping genes: β-actin (*Actb*) and β-2 microglobulin (*B2m*) and quantified by the 2^ΔΔct^ method (Livak and Schmittgen, 2001). RT2 qPCR Primer Assays (QIAGEN) were ordered with the following IDs: *Actb:* PPM02954B; *B2m:* PPM03562A; liver kinase B1 (*Lkb1*): PPM31360A; *Mef2c*: PPM04548A. To assess *Lkb1* gene expression in cells, RNA was extracted with the RNeasy Micro kit (QIAGEN), following the supplier's instructions. Then, 100 ng of RNA were reverse-transcribed to cDNA with the iScript gDNA Clear cDNA Synthesis Kit (172-5035 Bio-Rad Laboratories) following the manufacturer's instructions. qRT-PCR was performed on the CFX384 Connect Real-Time PCR System (Bio-Rad) using PrimePCR assays SYBR^®^ green (LKB1 Unique Assay ID: qMmuC ID0006462 Bio-Rad). For each experiment, samples were analyzed in technical triplicate. The mRNA expression of genes was normalized to the mean of two housekeeping genes, *Actb* (qMmuCED0027505 Bio-Rad) and *Hprt* (qMmuCID0005679 Bio-Rad) and quantified by the 2^ΔΔct^ method.

### Protein expression analysis by western blot

Protein extractions and immunoblots for the determination of targets of interest were performed in left GC muscles of different experimental groups. All muscles were homogenized in ice-cold buffer containing 20 mM Tris-HCl (pH 7.4 at 4°C), 2% SDS, 5 mM EDTA, 5 mM EGTA, 1 mM DTT, 100 mM NaF, 2 mM sodium vanadate, 0.5 mM phenylmethylsulfonyl fluoride, 10 mg/ml leupeptin and 10 ml/ml pepstatin. Homogenates were centrifuged at 1500 ***g*** for 10 min at 4°C and the supernatant was quantified using the BCA Protein Assay Kit (bicinchoninic acid solution, 1003278214; copper (III) sulfate solution, 1003274018; Sigma-Aldrich) according to the manufacturer's instructions. For immunoblot analysis, 20 μg of protein were separated on a 10% SDS-PAGE gel and transferred onto PVDF membrane for 7 min at 1.3 A–25 V (Trans-Blot^®^ Turbo™ Transfer System, Bio-Rad Laboratories); protein detection was conducted as described previously (Capogrosso et al., 2017; Mantuano et al., 2020). Anti-mouse (1:5000; anti-mouse IgG, A9044, Sigma-Aldrich) and anti-rabbit (1:5000; anti-mouse IgG, 170-6515, Bio-Rad Laboratories) horseradish peroxidase-conjugated secondary antibodies were used.

The following dilutions of primary antibodies were used: 1:400 mouse anti-vinculin (Sigma-Aldrich, V9131); 1:700 rabbit anti-LKB1 (Cell Signaling Technology, BK3047S); 1:700 rabbit anti-MO25α/CAB39 (Cell Signaling Technology, BK2716S); 1:300 rabbit anti LYK5 (STRADα) (Sigma-Aldrich, AV49077); 1:700 rabbit anti-HDAC4 (Cell Signaling Technology, BK15164S); 1:300 rabbit anti-HDAC5 (Cell Signaling Technology, BK20458S); 1:500 rabbit phospho-HDAC4 (Ser246)/HDAC5 (Ser259) (Cell Signaling Technology, BK3443S); 1:500 rabbit anti-AMPK (Cell Signaling Technology, 2532); 1:500 rabbit anti-phospho-AMPKα (Thr172) (Cell Signaling Technology, BK2531S); 1:400 rabbit anti-SIK2 (Cell Signaling Technology, 6919); 1:400 rabbit anti-phospho-SIK2 (Thr175, Thr163) (Invitrogen Thermo Fisher Scientific, PA5-64607).

LKB1, MO25α and STRADα levels were normalized to vinculin. p-AMPK and p-SIK2 were normalized to total AMPK and SIK2, detected on the same membrane after stripping with β-mercaptoethanol-containing stripping buffer (20% SDS, Tris-HCl 1 M pH 6.8, β-mercaptoethanol 0.1 M) for 30 min at 50°C. The membrane was washed in washing buffer (PBS 10%, Tween-20 0.1%), before being incubated with the next primary antibody. Because the pHDAC4/5 antibody does not discriminate the phosphorylated forms of HDAC4 (Ser246) and HDAC5 (Ser259) alone, we normalized pHDAC4/5 to the sum of total HDAC4 and HDAC5 detected on the same membrane after two subsequent strippings.

Densitometric analysis was performed using Image Laboratory software (Bio-Rad Laboratories). The software allows the chemiluminescence detection of each experimental protein band to obtain the absolute signal intensity. The density volume was automatically adjusted by subtracting the local background.

### Immunofluorescence analysis

Serial cross-sections (10 μm thick) from each frozen left GC muscle were transversally cut on a cryostat microtome set at −20°C (HM 525 NX, Thermo Fisher Scientific) and mounted on slides (SuperFrost^®^ Plus, Menzel Gläser, Thermo Fisher Scientific). 2B4 and SF1 cells were grown on plates at seeding density of 2.5×10^4^ for 11 days. Tissue sections and cells were fixed in paraformaldehyde for 15 min at room temperature (RT) and permeabilized with 0.1% TWEEN 20 (Sigma-Aldrich) and 0.1% bovine serum albumin (Sigma-Aldrich) in PBS for 45 min at RT. After three washes with PBS, sections and cells were blocked in saturation buffer (0.1% bovine serum albumin in PBS) and incubated with a rabbit polyclonal anti-laminin primary antibody (1:500; L9393S, Sigma-Aldrich) and mouse monoclonal anti-LKB1 primary antibody (1:50; sc-32245, Santa Cruz Biotechnology) for 2 h at RT in blocking buffer. After three washes in PBS, sections and cells were incubated with 488/594 Alexa Fluor-conjugated secondary antibodies (1:1000; A21206 and A32744, Thermo Fisher Scientific) for 45 min at RT. Nuclei were stained with PureBlu^TM^ Hoechst 33342 Nuclear Staining Dye (1:50; Bio-Rad). Images were captured using a dark-field microscope (CL-I Eclipse Nikon) at 20× magnification.

### Statistics

All experimental data were expressed as mean±s.e.m. Multiple statistical comparisons among groups belonging to different strains (WT, *mdx*, D2 WT and D2 *mdx*) was assessed by two-way ANOVA analysis, with Bonferroni's *t*-test post-hoc correction when the null hypothesis was rejected (*P*<0.05). Single comparisons between individual means were assessed by unpaired Student's *t*-test to analyze specifically differences due to genotype (*mdx* mice versus WT mice, D2 *mdx* mice versus D2 WT mice), exercise (exercised mice versus sedentary mice of the same genotype) or treatment (treated mice versus untreated mice of the same genotype). Statistical analyses were performed using GraphPad Prism version 8.
